# Grain Refinement Efficiency in Commercial-Purity Aluminum Influenced by the Addition of Al-4Ti Master Alloys with Varying TiAl_3_ Particles

**DOI:** 10.3390/ma9110869

**Published:** 2016-10-26

**Authors:** Jianhua Zhao, Jiansheng He, Qi Tang, Tao Wang, Jing Chen

**Affiliations:** 1 State Key Laboratory of Mechanical Transmission, College of Materials Science and Engineering, Chongqing University, Chongqing 400044, China; jiansheng.he@cqu.edu.cn (J.H.); 20150901003@cqu.edu.cn (Q.T.); 20113869@cqu.edu.cn (T.W.); 20150902010@cqu.edu.cn (J.C.); 2 National Engineering Research Center for Magnesium Alloys, Chongqing University, Chongqing 400044, China

**Keywords:** Al-4Ti, grain refinement, microstructure, morphology

## Abstract

A series of Al-4Ti master alloys with various TiAl_3_ particles were prepared via pouring the pure aluminum added with K_2_TiF_6_ or sponge titanium into three different molds made of graphite, copper, and sand. The microstructure and morphology of TiAl_3_ particles were characterized and analyzed by scanning electron microscope (SEM) with energy dispersive spectroscopy (EDS). The microstructure of TiAl_3_ particles in Al-4Ti master alloys and their grain refinement efficiency in commercial-purity aluminum were investigated in this study. Results show that there were three different morphologies of TiAl_3_ particles in Al-4Ti master alloys: petal-like structures, blocky structures, and flaky structures. The Al-4Ti master alloy with blocky TiAl_3_ particles had better and more stable grain refinement efficiency than the master alloys with petal-like and flaky TiAl_3_ particles. The average grain size of the refined commercial-purity aluminum always hereditarily followed the size of the original TiAl_3_ particles. In addition, the grain refinement efficiency of Al-4Ti master alloys with the same morphology, size, and distribution of TiAl_3_ particles prepared through different processes was almost identical.

## 1. Introduction

It is well known that metals and alloys usually solidify with coarse columnar grain structures under normal casting condition unless the details and process of solidification are carefully controlled [1,2]. Fine and equiaxed grains could be acquired by adding some master alloys into molten aluminum [3,4,5]. The cast alloys with equiaxed grain structure have high toughness, high yield strength, excellent formability, good surface finish, and improved ability, achieving a uniform anodized surface, better fatigue life, and good machinability. Thus, there is a quite a need to prepare and develop this type of high performance cast alloy [6,7]. Moreover, a sound grain practice has the following advantages: the avoidance to hot tearing, the allowance for an increase of the casting speed, and the improvement of the casting structure homogeneity by redistribution of the second phases and micro porosity [4,8]. At present, the addition of Al-Ti-B master alloy into the aluminum melt is a common method for grain refinement in industry. Compared with the Al-Ti-B master alloy of high efficiency, the Al-Ti master alloys are considered to be simple and are easily ignored [9,10,11]. However, the Al-Ti master alloys have been frequently used as a substitute for the Al-Ti-B master alloy in some specific areas, such as aluminum foil, electronic accessories, etc.

There is no doubt that TiAl_3_ particles in Al-Ti master alloys act as the centers of heterogeneous nucleation in the mechanism of grain refinement [12]. The morphology, size, and number of the nuclei seem to play important roles in determining the grain refinement efficiency of the master alloys [12,13,14,15]. The preparation process and grain refinement performance of Al-Ti and Al-Ti-B master alloys have been investigated in some studies. Those studies mainly focused on the influence of various compositions [16] and processing parameters, such as hold time [17], cooling rate [18], reaction temperature [17,19], and stirring conditions [20] on the grain refinement efficiency. Additionally, most studies paid less attention to the effect of the cooling rate on morphology of TiAl_3_ particles in Al-4Ti alloys, and the grain refinement performance of petal TiAl_3_ particles has never been mentioned in the studies. Therefore, the formation process of various TiAl_3_ particles in Al-4Ti master alloys and their grain refinement performance in commercial-purity aluminum were investigated in this paper.

## 2. Experimental

The Al-4Ti master alloys were acquired by adding K_2_TiF_6_ powders or spongy titanium into commercially-pure aluminum in an electrical resistance furnace at 850 °C. A series of Al-4Ti master alloys with various TiAl_3_ particles were prepared through different processes as shown in Table 1. The specimens were taken from the master alloys and glazed by mechanical and electrochemical polishing. For further observation, a solution of 0.5% hydrofluoric acid (HF) was used to lightly etch all of the specimens. Then the samples were deeply etched in the 10% NaOH-distilled water solution for 10 min at 60 °C. The microstructures and three-dimensional morphologies of TiAl_3_ particles in Al-4Ti alloys were characterized by using TESCANVEGA II scanning electron microscope (SEM) (TESCAN, Bruno, Czech Republic) with light element energy dispersive spectroscopy (EDS) X-ray detectors.

**Figure 1:**
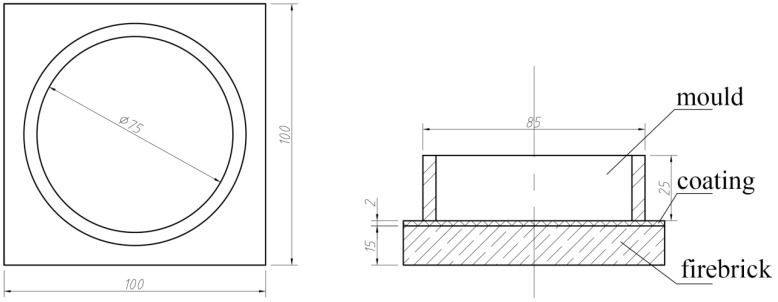
Schematic illustration of the mold for grain refinement evaluation.

To evaluate the grain refinement performance, the master alloys I, II, III, IV, V, and VI, which occupied about 0.2 wt. % of the whole melts, were independently added into the commercial-purity aluminum ingots at 720 °C, which was proved to be the temperature with the highest recovery of titanium [17]. The melts were kept in the resistance furnace for 5, 10, 30, 60, and 120 min, respectively, and then poured into a ring-like mold made of steel. The shape and other details of the mold were shown in Figure 1. The samples were directly etched by the mixed acid reagent (87.5% HCl + 10% HNO_3_ + 2.5% HF). Finally, the grain size of the refined samples were observed and analyzed by a Carl Zeiss Microscopy (GmbH 37081, Göttingen, Germany).

## 3. Results and Discussion

### 3.1. Microstructures and Morphologies

**Figure 2:**
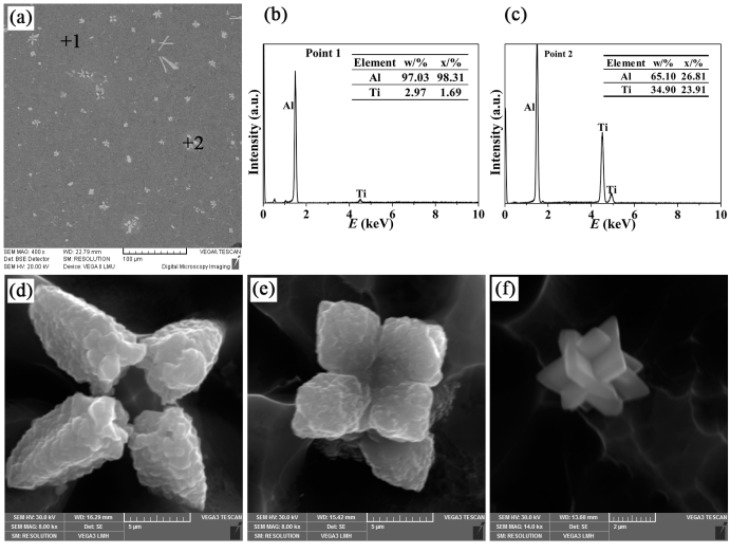
(**a**) SEM micrograph of the Al-4Ti master alloy I; (**b**,**c**) EDS spectra of points 1 and 2 marked in (**a**), respectively; and (**d**–**f**) three-dimensional morphologies of the petal-like TiAl3 particles.

**Figure 3:**
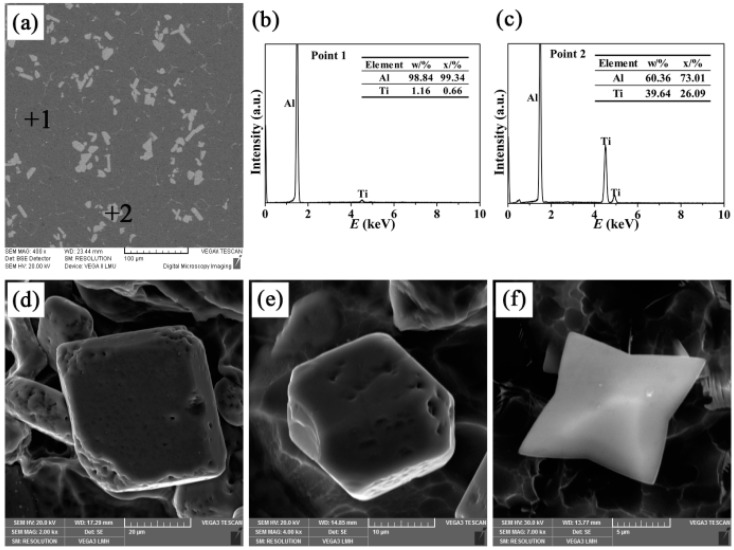
(**a**) SEM micrograph of the Al-4Ti master alloy II; (**b**,**c**) EDS spectra of points 1 and 2 marked in (**a**) respectively; and (**d**–**f**) three-dimensional morphologies of the blocky TiAl_3_ particles.

**Figure 4:**
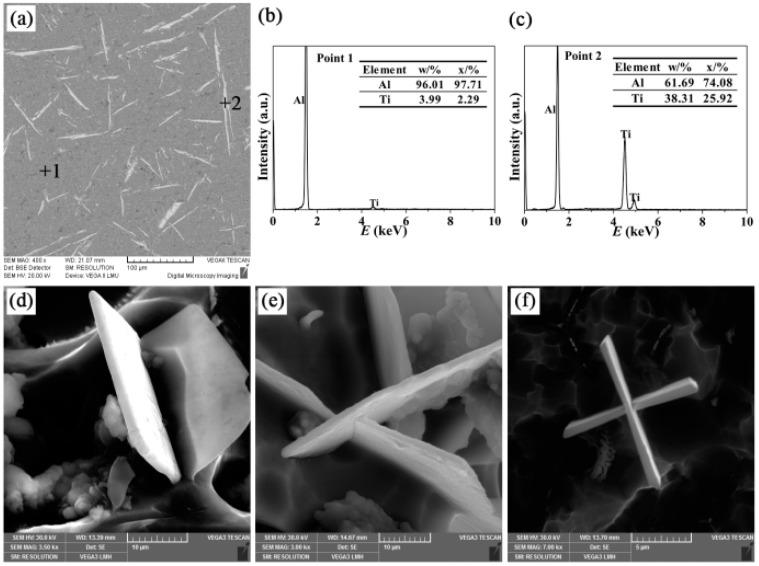
(**a**) SEM micrograph of the Al-4Ti master alloy III; (**b**,**c**) EDS spectra of points 1 and 2 marked in (**a**) respectively; and (**d**–**f**) three-dimensional morphologies of the flaky TiAl_3_ particles.

The microstructures of Al-4Ti master alloys (I, II, and III) and the three-dimensional morphologies of TiAl_3_ particles are shown in Figure 2, Figure 3 and Figure 4, respectively. It can be found that the TiAl_3_ intermetallic particles had three different morphologies: petal-like structures, blocky structures, and flaky structures, the formations of which have a very tight connection to the additions and mold materials. This is in agreement with the reports from Arnberg et al. [21] and Liu et al. [22]. The petal-like TiAl_3_ particles tend to form at low melting temperatures and high cooling rates. The blocky TiAl_3_ particles are more likely to generate at low melt temperatures and medium cooling rates, while the flaky TiAl_3_ particles are inclined to grow at high melt temperatures and low cooling rates.

Figure 2 shows the microstructure of Al-4Ti master alloy I and the three-dimensional morphologies of petal-like TiAl_3_ particles. According to Figure 2a–c, it can be observed that petal-like TiAl_3_ particles distribute uniformly on the aluminum substrate, which can also be found in the study of Arnberg et al. [23]. Figure 2d–f exhibit the three-dimensional morphologies of petal-like TiAl_3_ particles in Al-4Ti master alloy I. Based on Figure 2d, it could be deduced that the free-distributed TiAl_3_ particles were treated as nucleation centers, from which all of the petals started to grow. During the whole process, Ti atoms had to be diffused and transported a long distance to fill the deficiency of titanium for marginal growth and, thus, the growth rate of petals would decrease as the cooling time increased. It was not until the adjacent petals contacted with each other that the petal-like TiAl_3_ particles would stop growing. Due to the change of growth rate, the TiAl_3_ particles in the Al-4Ti master alloy I were in the shape of petals with cusps. In Figure 2f, it could be found that the growth of TiAl_3_ particles might conform to a twinning mechanism. With the decrease of the melt temperature, the undercooling between (001) and the melt interface increased to a new level, which was not enough to form a new phase. On the basis of the twinning growth mechanism, a new plane formed and grew at the intersection of the new and old c-axes, which were mutually perpendicular [18]. Until the titanium at the margin was run out, the petals would stop growing. After that, the growth of TiAl_3_ would follow the twinning mechanism. As a result, the petal-like TiAl_3_ particles were formed in Al-4Ti master alloy I.

Figure 3 displays the microstructure of Al-4Ti master alloy II and the three-dimensional morphologies of blocky TiAl_3_ particles. In accordance with the EDS spectra in Figure 3b,c, it reveals that these white blocks were TiAl_3_ particles and the dark substrate was aluminum. As shown in Figure 3d–f, the TiAl_3_ particles present a tetrahedral or hexahedral structure, which also existed in Al-Ti and Al-Ti-C master alloys after rapid solidification [18,24,25]. This confirms the results of Wang et al. [26] that TiAl_3_ particles present a tetragonal structure. When titanium content was super-saturated in the melt, the blocky TiAl_3_ particles would form. In this case, the nuclear driving force of TiAl_3_ was enough to support the nucleation on the densest atomic plane of the original crystal, and the growth rate in the direction of [001] could not be ignored [27]. The influence of Ti atom diffusion was so little that the tendency of preferred growth was not also conspicuous. Therefore, the growth rates of all crystal planes were equalized and the blocky TiAl_3_ particles could be formed in the Al-4Ti master alloy II.

Figure 4 exhibits the microstructure of Al-4Ti master alloy III and the three-dimensional morphologies of flaky TiAl_3_ particles. The TiAl_3_ particles were in needle-like structures in two dimensions and formed flaky structures in three dimensions. The existence of TiAl_3_ particles could be confirmed by EDS analysis, as shown in Figure 4c. It could be observed from Figure 4d,e that there were some embossments on the surfaces of flaking. In addition, Figure 4e shows that the aspect ratio of the flaky TiAl_3_ particles is larger than the blocky ones, which indicates that the growth of these flaky particles was influenced by strain energy. Lee et al. proposed an explanation that the flaky particles are formed in the solid phase by precipitation from a supersaturated solid solution of titanium in an aluminum melt [28]. The flaky TiAl_3_ intermetallic compounds were two-dimensional dendrite crystals, which meant that the growth rates in the [100], [010], and [110] directions were identical. However, the (001) crystal plane had the maximum atomic density, and the growth rate in the [001] direction was the lowest [26]. Finally, the flaky TiAl_3_ particles were formed in Al-4Ti master alloy III, which is also consistent with the forecast of Flemings [2].

The microstructural parameters of different Al-4Ti master alloys prepared in the experiment are shown in Table 2. When K_2_TiF_6_ was added into the commercial-purity aluminum, the morphology of TiAl_3_ particles experienced a transformation from petal-like structures to blocky structures, and then to flaky structures as the cooling rate decreased. At high cooling rates, the solidification time was too short to support growth and, thus, the majority of petal-like TiAl_3_ particles were tiny. By comparison, when the commercial-purity aluminum added with the sponge titanium was cast into all three kinds of molds, there was no petal-like TiAl_3_ particle in the Al-4Ti master alloys. In addition, the TiAl_3_ particles in Al-4Ti master alloys IV and V displayed a blocky structure but had different sizes. This means that not only the morphologies of TiAl_3_ particles in Al-4Ti master alloys, but also the grain sizes of the blocky particles, were deeply related to the cooling rate. In general, the petal-like TiAl_3_ particles were only formed when the aluminum melt augmented with K_2_TiF_6_ was casted into the graphite mold. On one hand, the addition of K_2_TiF_6_ could distribute uniformly in the aluminum melt, while the addition of sponge titanium would sink and aggregate to produce some titanium super-saturation areas, which might not be beneficial to the formation of petal-like TiAl_3_ particles. On the other hand, the addition of K_2_TiF_6_ may cause the alteration of the preferential growth orientation of TiAl_3_ crystals. Furthermore, the grain size of different TiAl_3_ particles in Al-4Ti master alloys increased gradually with the decrease of the cooling rate within the same additions.

### 3.2. Refinement Performance of Al-4Ti Master Alloys

**Figure 5:**
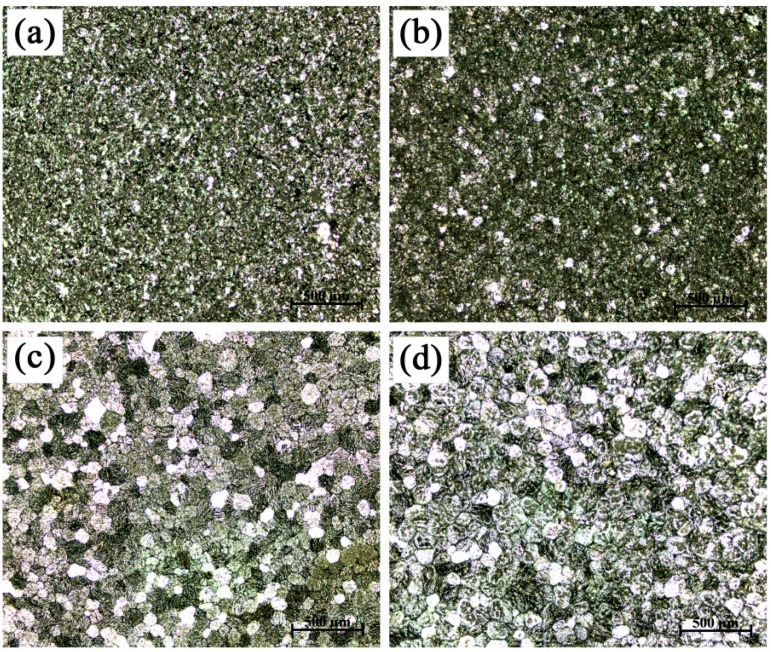

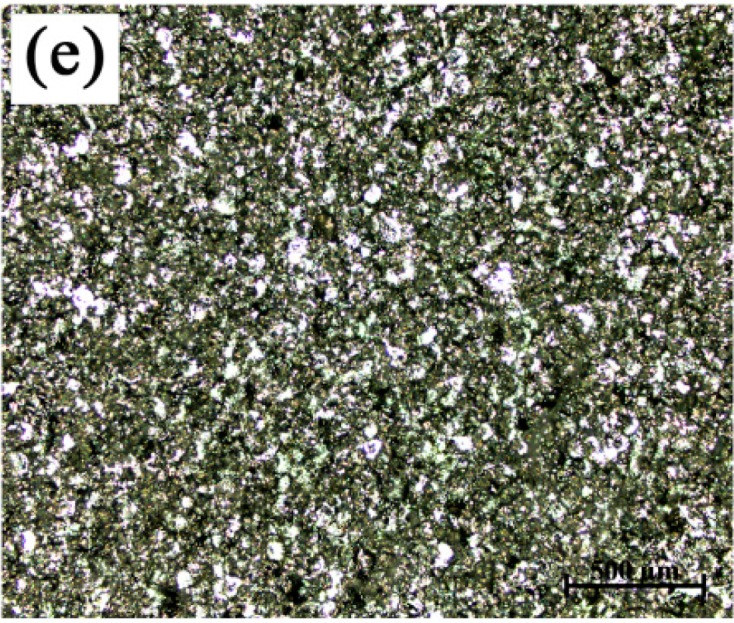
Macrostructures of commercial-purity aluminum sample refined by Al-4Ti master alloy I (0.2 wt. %) after different hold times: (**a**) 5 min; (**b**) 10 min; (**c**) 30 min; (**d**) 60 min; and (**e**) 120 min.

**Figure 6:**
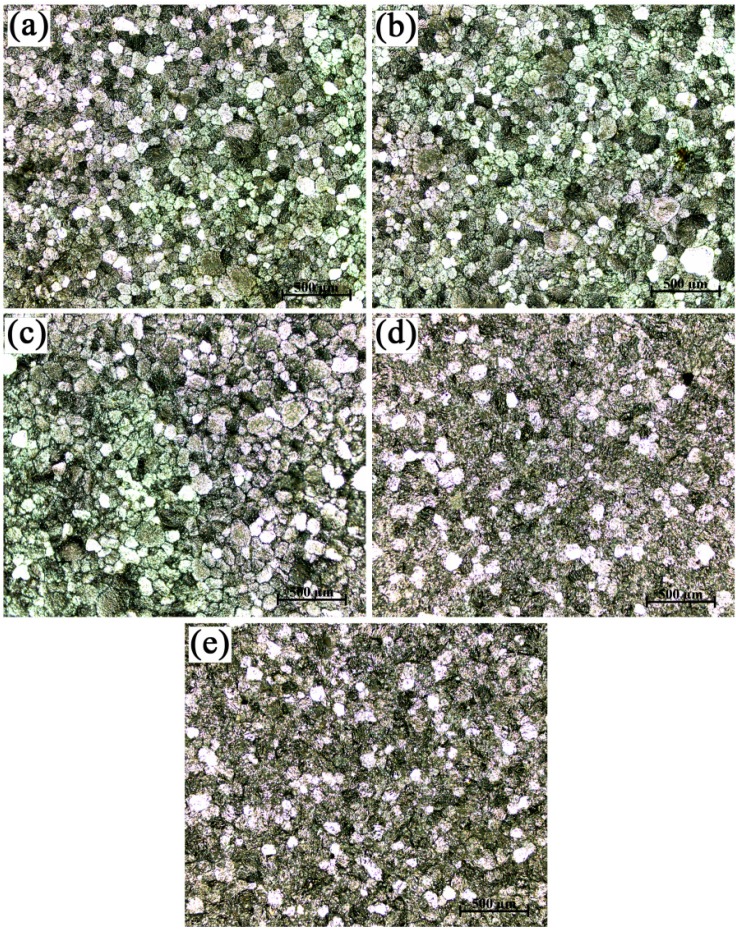
Macrostructures of commercial-purity aluminum sample refined by Al-4Ti master alloy II (0.2 wt. %) after different hold times: (**a**) 5 min; (**b**) 10 min; (**c**) 30 min; (**d**) 60 min; and (**e**) 120 min.

**Figure 7:**
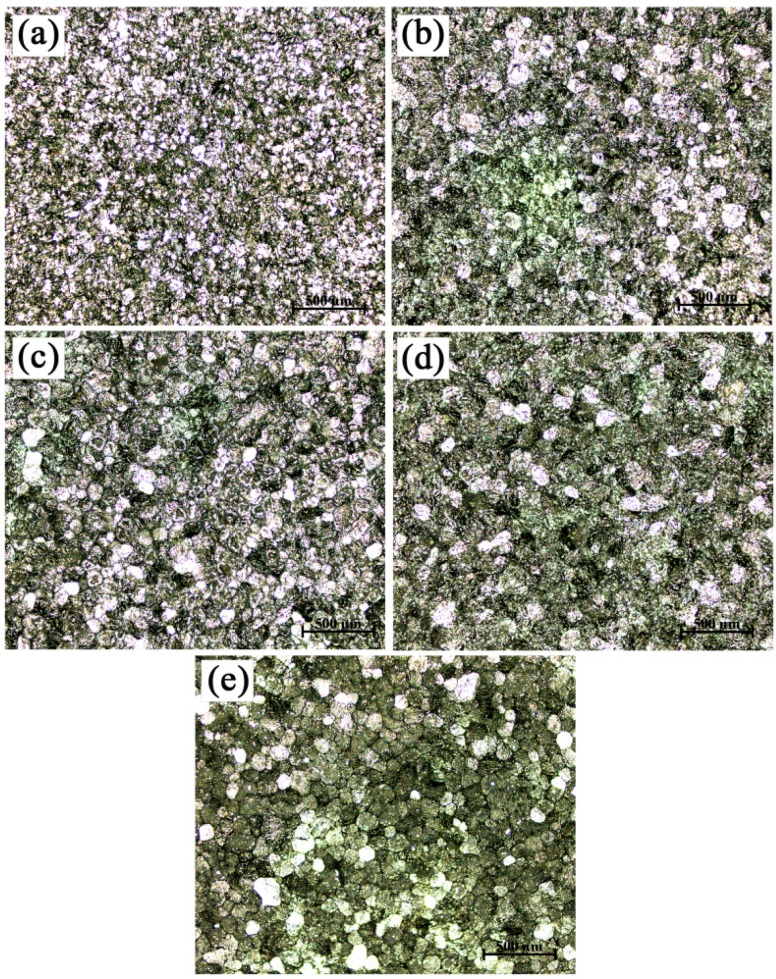
Macrostructures of commercial-purity aluminum sample refined by Al-4Ti master alloy III (0.2 wt. %) after different hold times: (**a**) 5 min; (**b**) 10 min; (**c**) 30 min; (**d**) 60 min; (**e**) 120 min.

Additional experiments were employed to evaluate the effects of hold time on the refinement efficiency of Al-4Ti master alloys with different types of TiAl_3_ particles in commercial-purity aluminum. The macrostructures of commercial-purity aluminum refined by Al-4Ti master alloys I, II, and III, respectively, were shown in Figure 5, Figure 6 and Figure 7. There existed many differences of grain refinement efficiency among the various types of Al-4Ti master alloys. After comprehensive analysis of all of the hold times, the grain refinement efficiency of the Al-4Ti master alloy I with petal-like TiAl_3_ particles decreased gradually, and then increased sharply, as shown in Figure 5. The grain size of commercial-purity aluminum reached the maximum when the hold time was 60 min. Additionally, both the grain size of commercial-purity aluminum refined for 5 min and 120 min was about 65 μm. The results in this study reach agreement with the discoveries of Li et al. [13] regarding the grain refinement performance of the Al-4Ti master alloy with both flaky and blocky TiAl_3_ particles. When Al-4Ti master alloy I was added into commercial-purity aluminum, the free petal-like TiAl_3_ particles worked as the nuclei, leading to an improvement of grain refinement efficiency. Some TiAl_3_ particles formed according to the repeated twinning mechanism might break into small flakes to provide more nuclei, which could also promote the grain refinement efficiency. However, some petal-like TiAl_3_ particles (Figure 2f) dissolved gradually, and the number of heterogeneous nuclei would reduce, which caused the further growth in grain size. Moreover, some petal-like TiAl_3_ particles (Figure 2d,e) would break into small blocks which could prevent the small flakes mentioned before from dissolving. Therefore, it could be deduced that the various types of TiAl_3_ particles could transform into each other, and the blocky TiAl_3_ particles could effectively prevent the small flakes, decomposed by the petal-like particles, from dissolving.

As can be seen from Figure 6, the grain refinement efficiency of the Al-4Ti master alloy II with blocky TiAl_3_ particles was the best and did not fade away with the change of the hold time. The grain refinement performance of the Al-4Ti master alloy with blocky TiAl_3_ particles was better than that of the master alloy I with petal-like TiAl_3_ particles. As for the master alloy III with flaky TiAl_3_ particles, the conversion from coarse columnar grains to fine equiaxed grains was completed within 2 min (Figure 7). The grain refinement performance of the master alloy III with flaky TiAl_3_ particles would worsen as the hold time increased. The grain size of commercial-purity aluminum reached the maximum when the hold time was 120 min. This was because the dissolution of all of the flaky TiAl_3_ particles, with the increase of hold time, provided fewer nuclei.

**Figure 8:**
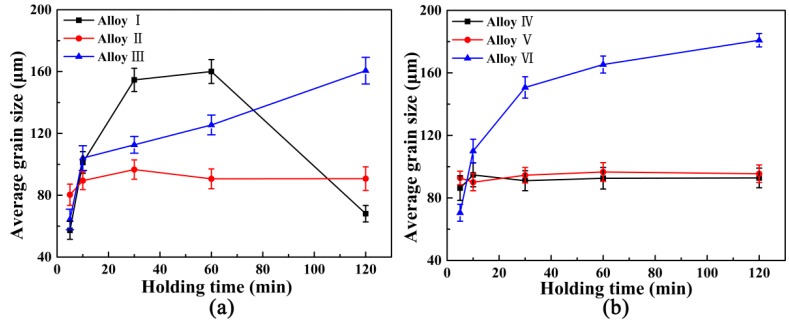
Relationships between average grain size and hold time: (**a**) the Al-4Ti master alloys I, II, and III; and (**b**) the Al-4Ti master alloys IV, V, and VI.

Figure 8 shows the relationships between average grain size and hold time of the Al-4Ti master alloys I, II, III, IV, V, and VI. It was found that the grain refinement efficiency of the master alloy I with petal-like TiAl_3_ particles decreased and then strengthened. For the master alloys III and VI with flaky TiAl_3_ particles, the grain size of commercial-purity aluminum went up with the increase of the hold time. The three curves of the master alloys II, IV, and V with blocky TiAl_3_ particles remained level and stable. Therefore, the grain refinement efficiency of the master alloys with blocky TiAl_3_ particles was better than the master alloys with petal-like and flaky TiAl_3_ particles under the same condition of addition. The grains in commercial-purity aluminum after grain refinement of Al-4Ti master alloys also showed a hereditary effect of the original grain size. The average grain size of the refined commercial-purity aluminum always hereditarily followed the size of the original TiAl_3_ particles. In addition, it could be also deduced that no matter what the preparation process was, only the grain refinement efficiency of Al-4Ti master alloys with the same morphology, size, and distribution of TiAl_3_ particles was almost the same.

## 4. Conclusions

In this work, the formation process of various TiAl_3_ particles in Al-4Ti master alloys and their grain refinement performance in commercial-purity aluminum were studied. The major findings are as follows:

(i) There were three different types of TiAl_3_ particles in Al-4Ti master alloys: petal-like structures, blocky structures, and flaky structures. The petal-like TiAl_3_ particles were only formed according to the growth mechanism of repeated twins when the aluminum melt augmented with K_2_TiF_6_ was casted into the graphite mold. Whether the K_2_TiF_6_ or sponge titanium was added into the aluminum melt which was cast into the sand mold, the flaky TiAl_3_ particles were generated. In addition, the blocky TiAl_3_ particles were found after the remaining three different processes.
(ii) The grains in commercial-purity aluminum after grain refinement of Al-4Ti master alloys showed a hereditary effect on grain size. The larger the average grain sizes of the original TiAl_3_ particles were, the larger that of the refined commercial-purity aluminum was.
(iii) With the increase in the hold time, the grain size of the commercial-purity aluminum refined by the Al-4Ti master alloy with petal-like TiAl_3_ particles increased, at first, and then decreased rapidly. The grain refinement efficiency of the Al-4Ti master alloy with blocky TiAl_3_ particles was the best and did not fade away with the change of the hold time. The Al-4Ti master alloy with flaky TiAl_3_ particles could instantly achieve a fine grain refinement under a hold time of five minutes; however, the fine refinement efficiency went down as the hold time increased.
(iv) The grain refinement efficiency of Al-4Ti master alloys with the same morphology, size, and distribution of TiAl_3_ particles prepared through different processes was almost identical.

## Figures and Tables

**Table 1:** Process details of the Al-4Ti master alloys.

Cast Material	Addition	Mold Materials	Alloy Designation
Pure aluminum	K_2_TiF_6_	Graphite	I
		Copper	II
		Sand	III
	Sponge titanium	Graphite	IV
		Copper	V
		Sand	VI

**Table 2:** Microstructural parameters of TiAl_3_ particles in Al-4Ti master alloys.

Alloy	Morphology	Average Length, μm	Quantity, cm^2^
I	Petal-like	11	24,340
II	Blocky	16	20,411
III	Needle-like	90	7462
IV	Blocky	13	23,750
V	Blocky	22	17,900
VI	Needle-like	106	6358
